# Exoskeleton kinematic design robustness: An assessment method to account for human variability

**DOI:** 10.1017/wtc.2020.7

**Published:** 2020-11-04

**Authors:** Matteo Sposito, Christian Di Natali, Stefano Toxiri, Darwin G. Caldwell, Elena De Momi, Jesús Ortiz

**Affiliations:** 1 Advanced Robotic (ADVR), Istituto Italiano di Tecnologia, Genova, Italy; 2 Dipartimento di Elettronica, Informazione e Bioingegneria (DEIB), Politecnico di Milano, Milano, Italy

**Keywords:** Exoskeletons, Design, Performance characterisation, Human in the loop optimisation, Industry

## Abstract

Exoskeletons are wearable devices intended to physically assist one or multiple human joints in executing certain activities. From a mechanical point of view, they are kinematic structures arranged in parallel to the biological joints. In order to allow the users to move while assisted, it is crucial to avoid mobility restrictions introduced by the exoskeleton’s kinematics. Passive degrees of freedom and other self-alignment mechanisms are a common option to avoid any restrictions. However, the literature lacks a systematic method to account for large inter- and intra-subject variability in designing and assessing kinematic chains. To this end, we introduce a model-based method to assess the kinematics of exoskeletons by representing restrictions in mobility as disturbances and undesired forces at the anchor points. The method makes use of robotic kinematic tools and generates useful insights to support the design process. Though an application on a back-support exoskeleton designed for lifting tasks is illustrated, the method can describe any type of rigid exoskeleton. A qualitative pilot trial is conducted to assess the kinematic model that proved to predict kinematic configurations associated to rising undesired forces recorded at the anchor points, that give rise to mobility restrictions and discomfort on the users.

## 1 Introduction

Exoskeletons are wearable devices that can physically assist one or multiple biological joints. These devices can be regarded as an additional kinematic chain parallel to the corresponding biological one. They aim to augment users’ physical capabilities to reduce the load on their joints, or to compensate for impaired muscles. They have been developed and specialized to provide assistance in military, occupational, medical, and rehabilitation applications.

**Figure 1.**
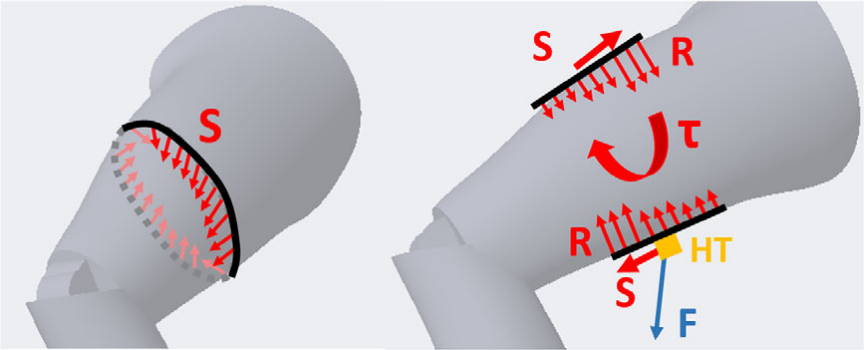
Undesired reaction forces and torques exerted on limbs by misalignments. On the left radial, Shear forces created harnesses and garments. On the right longitudinal Shear and reaction Torques over the limb caused by braces through the attachment point HT where the rigid exoskeleton connects to the body.

In particular, occupational exoskeletons have the potential to improve wellness and safety of an ageing working class (UNI Global Union Europe, 2015). Growing interest in these devices is supported by scientific assessment of their effectiveness (de Looze et al., 2016; Nussbaum et al., 2019; Theurel and Desbrosses, 2019), the emergence of shared evaluation indexes (Pietrantoni et al., 2019; Torricelli and Pons, 2019), and reports and guides to inform users (i.e., ergonomic practitioners, workers, and customers) (Sugar et al., 2018; Toxiri et al., 2019). Despite the increasing interest, acceptance rates of wearable augmenting devices, especially for healthy subjects, is influenced by several factors and it is closely related to design solutions affecting both physical and mental load imposed by the exoskeleton (e.g., added inertia and weight or non-intuitive control strategies; Stirling et al., 2019). Indeed, an exoskeleton is meant to assist the physical capabilities of subjects but this function must not be invalidated by physical discomfort and mental stress, ultimately leading to a possible rejection of the device. Regarding physical discomfort, exoskeletons may hinder the user in different ways. Designers can decrease weight or motor inertia but exoskeleton efficiency and user comfort are intimately connected to the device kinematics, physical attachments (defined as braces for exoskeletons), and their stability over time. Anthropomorphic exoskeletons joints must be aligned and match the human ones or implement some sort of alternative mechanism to avoid incompatibilities. Näf et al. (2019) and Schiele (2008) analyze the causes, effects, and common solutions to such incompatibilities. These are mostly represented by the misalignment of the exoskeleton’s joints instantaneous centre of rotation (ICR) with respect to the desired position. Causes of human and exoskeleton ICR’s misalignments are drifts relative to each other during motion, initial fitting mismatch, and anthropometric variations within the targeted population. Misalignments result in undesired forces and torques exerted on the body through braces, part of which could reduce assistance efficacy and generate parasitic reaction forces. Redundant and passive degrees of freedoms (DOFs) and manual regulation mechanisms are the most common solutions to avoid these parasitic effects of kinematic mismatches (Cempini et al., 2013). Anchor point compliance is another viable solution to reduce the discomfort of the device. Compliance at fixation points is, of course, added by human soft tissues but it can be additionally increased by elastic and flexible materials in the braces (Näf et al., 2019). However, any self-alignment mechanism (SAM) or compliant parts can only absorb misalignments negative effects within a specific range and outside this range, parasitic forces will appear. Parasitic forces and torques will often be unpredictable in amplitude and direction, and when unloading on fixations can result in shear over the skin and movement of the attachments. Further drift of attachment points will make the situation even worse. The effects of misalignments on the body also depends on the type and position of braces. Cuffs and orthoses are the rigid part of braces and tend to create reaction torques on limbs rather than forces (Figure 1). This results in torsional shear around the limb axis (Jarrassé and Morel, 2012). Soft tissue shear and compression can occur over localized but wide body areas. On the contrary, the textile part of the braces, garments, and harnesses, do transmit reaction forces resulting in shear along limb axis. Shear and compression can occur over multiple body areas behaving like a torniquet (Kermavnar et al., 2017).

### 1.1 Design pipelines

Since exoskeletons can be considered as wearable robots, designers often use the same tools and design methodology as for conventional robots: defining requirements (such as reachable workspace, weight, and type of actuation), designing and developing the exoskeleton, undergoing user testing to extract feedback and iterating to refine the design. However, designers of wearable robots must face specific challenges in connecting a hard mechanical structure to a human being and the conventional approach is not always ideal or even suitable. With this work, and the design of wearable devices in general, we aim to facilitate the involvement of the end users and implementation of their requirements, as early as in the design phase as possible. This is the fundamental part of a process called user-centered design (UCD) (O’Sullivan et al., 2017), that represent the methodology we want to adopt for the design of our test platform exoskeleton. To implement a participatory design approach, like UCD, it is of paramount importance to involve and include the final users with any mean: workshops, interactive media, collaborative design, or rewards (Binder et al., 2019). So far, few design guidelines have been presented, most of the literature reports cases where designers face a single specific issue (e.g., minimizing contact forces, movement hindering, or center of mass positioning) affecting device comfort.

Schiele and Van Der Helm (2006) addressed the problem of minimizing internal reaction forces for an upper limb rehabilitation exoskeleton. Those reaction forces are created by misalignments between the robotic and human kinematic chain. Starting from simplified biomechanical models (link-segment model [LSM]) of the shoulder and upper arm, they computed the reachable workspace of anchor points on the arm body segments in order to avoid kinematic singularities during the use of the exoskeleton. By applying a typical trajectory, based on the rehabilitation regime, active and passive DOFs were selected. In this way, also the self-alignment properties between the robot and the biological joints were ensured. This work is a good example of integrating human body kinematics into the design process, however, their target exoskeleton imposes a trajectory on the impaired body segment and little to no positioning variations on the braces are allowed. Jarrasse and Morel (2010) presented an analytical method to achieve user safety and comfort while avoiding hyperstaticity. They developed an analytical synthesis framework to assist designers to select the right DoFs configuration. Their work aims to optimize each joint’s range of motion (RoM) while the device and human limbs are coupled together. The framework returns several non-hyperstatic configurations for every closed-loop chain created by the attachments’ links, human limbs, and exoskeleton’s links. Jarrasse and Morel (2010) assumed that the anchor points are rigidly coupled with the body, and no movements are considered. Nonetheless, despite presenting valid and useful design criteria, none of the above approaches considered the consequences of inter- and intra-subject body segments variations. These differences would alter the required RoM of any misalignment compensation mechanism and, ultimately, can make a passive DoF useless. Moreover, in the real scenario, it is difficult to ensure perfect joint alignment between the exoskeleton and human. Thus, the exoskeleton design should be robust enough to ensure the same efficiency even if worn improperly.

Cempini et al. (2013) addressed the robustness of the exoskeleton’s design to fit problems by introducing variations in body kinematics during the design phase. They proposed an analytical framework that models body link’s ICR movements and drifts in time with additional joints. The framework output is a SAM, a redundant kinematic chain. The SAM was tested to assess the ability to transfer assistive torques to body segments and withstand a maximum tolerable 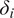 misalignments in any human joint. The study presented a methodology that can evaluate and synthesize a SAM with mathematical tools.

### 1.2 Contribution of this work

**Figure 2.**
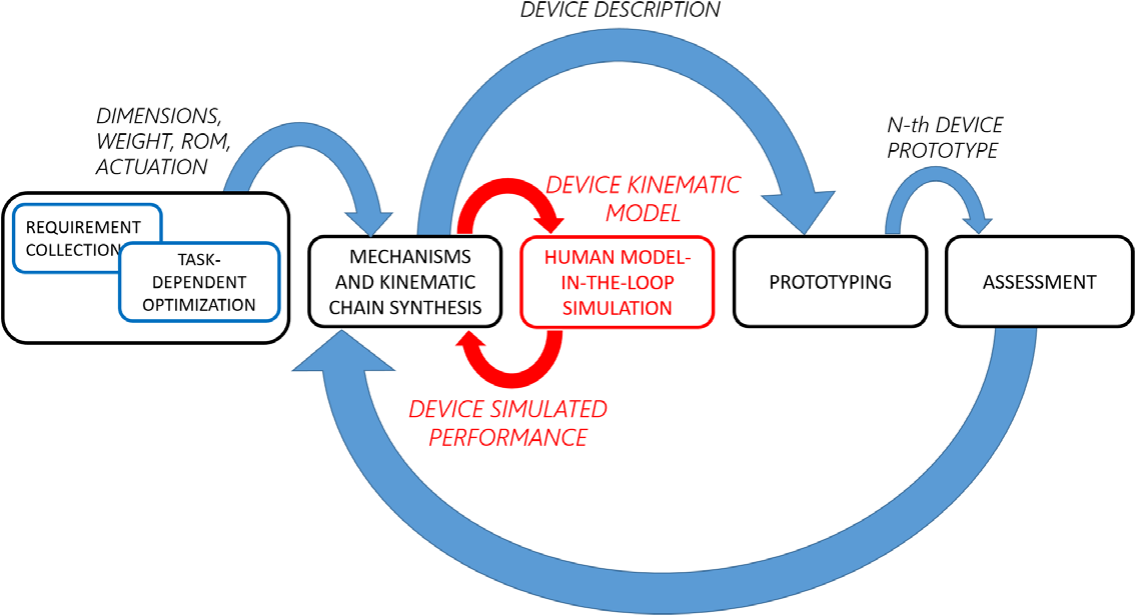
Typical development pipeline for exoskeleton design (Schiele and Van Der Helm, 2006; Jarrassé and Morel, 2012; Cempini et al., 2013). In red the human Model-in-the-Loop (MIL) step described in this paper.

This paper presents two major contributions: a *methodological contribution* and an *experimental contribution*. The *methodological contribution* consists in a simulation method to address, in the early development stage, ICR’s misalignments in exoskeleton kinematic design (introduced by anthropometry variations among users, fitting offset, and drift during motion). The simulation will evaluate and test the effects of modifications to braces’ (belts, straps, garments, or cuffs) positions resulting from induced motion or due to fit issues. The developed pipeline implements a MIL paradigm to test the design solutions in a simulated environment with data collected from the real part of the system (Plummer, 2006). The human MIL paradigm uses a healthy human subject’s anthropometrics data, kinematics, and RoM, as the real part and to compute the reactions of the simulated part of the model, the exoskeleton. This approach, in contrast to that proposed by Cempini et al. (2013), uses a MIL paradigm and group the source of position disturbance on attachments points. This method can be used for any type of rigid exoskeleton and requires a standard mathematical description, from robotics, of the human body and device (i.e., Denavit–Hartenberg parameters and joints’ RoM). Figure 2 shows the proposed pipeline and how this enhances the methods reported in the section “Design pipelines” The MIL simulation step, introduced in the section “Methods,” exploits traditional robotics tools and evaluation techniques such as Direct (DK) and Inverse Kinematic (IK) algorithms, Manipulability index (MI) (Yoshikawa, 1985), *Reachability* (Guan and Yokoi, 2006) and *Capability* (Zacharias et al., 2007) to show the exoskeleton’s SAM robustness to variations, caused by fit issues and users’ movements. As a part of the *methodological contribution,* we additionally introduce *Reachability* and *Capability* indexes for exoskeletons, to visually represent two different exoskeleton’s characteristics: Primary Workspace and Dexterity and their changes in different simulations. Primary Workspace is defined as the workspace calculated eliminating all the joints’ axis that pass through the robot end effector’s tip (Gupta, 1986). Dexterity is defined as the number of different tasks that the robot can perform or how well it can perform them (Ma and Dollar, 2011). In this work, we will consider the latter definition of Dexterity. *Reachability* and *Capability* are plotted in a discretized Cartesian tridimensional space (i.e., partitioned in *voxels*) to drastically reduce the required computational time. *Reachability* is introduced by Zacharias et al. (2007) and expanded by Diankov (2010) and we will use it to answer the question “can the exoskeleton follow all of the users’ positions?” and it can be useful to optimize the exoskeleton’s workspace (EW) shape with respect to the leg’s one. *Reachability* maps show space regions where the EW and leg’s workspace (LW) overlap. The map’s values are calculated over the primary workspaces of both exoskeleton and leg. However, *Reachability* does not provide information related to task-specific user’s movements. *Capability* is introduced by Zacharias et al. (2007) and we will use it to answer the question “how well the exoskeleton follow the users’ movements?” *Capability* maps show the dexterity of the exoskeleton in a compact spatial representation. The exoskeleton’s dexterity is calculated as End Point (EP) tip pose error with respect to the mechanical attachment point HT on the users’ leg (in Figure 1). The *Capability* index is calculated from real, or realistic, users’ movements that are performed during working tasks when the exoskeleton is supposed to be worn (i.e., walking, squatting, and stooping). The *experimental contribution* consists in a qualitative preliminary assessment of the proposed simulation method. The simulation and the qualitative assessment use the same model of exoskeleton test platform worn by different subjects. In addition, we introduce a new application of inertial sensing techniques to record exoskeleton’s drifts over the desired body fixation point. Drifts can occur during user’s movements and optical body capture techniques could not be applied because of marker’s occlusion.

In this paper, the MIL simulates only exoskeleton and body kinematics while dynamic and kinetic effects are neglected. The expected output, along with the visual help of Reachability and Capability maps, will guide mechanical parameters and design solutions to enhance comfort, safety, and efficiency of the selected device.

This paper is structured as follows: “Methods” section presents the kinematic model used in the MIL simulations and the considerations on where to apply perturbations. It provides a detailed description of the simulation algorithm in the section “Algorithm” and how to obtain an informational output in the sections “Reachability” and “Capability.” In “Experimental Validation” section, the rationale and experimental protocol of the MIL simulation and kinematic models validation are presented. Then, in “Results” section, we describe simulated and recorded data and different methods to visualize and interpret data. “Discussion” section presents comments on the obtained results and the correlation of the simulation outputs with the unwanted reaction forces recorded during the trials. “Limitations and future works” section provides the limitations of the presented model and its future development. Finally, “Conclusion” section provides for the final remarks of the work.

## 2 Methods

In this section, we present a MIL simulation to visualize the exoskeleton’s local and global dexterity. The simulation is computed for different attachment point’s position, that is moved because of inter- and intra-subject variability. We introduce the exoskeleton test platform and the simulation steps to obtain and define new Reachability and Capability indices for the device, using the same starting definitions set by Zacharias et al. (2007) and Porges et al. (2015) but applied to wearable robots.

### 2.1 Back-Support Exoskeleton

**Figure 3.**
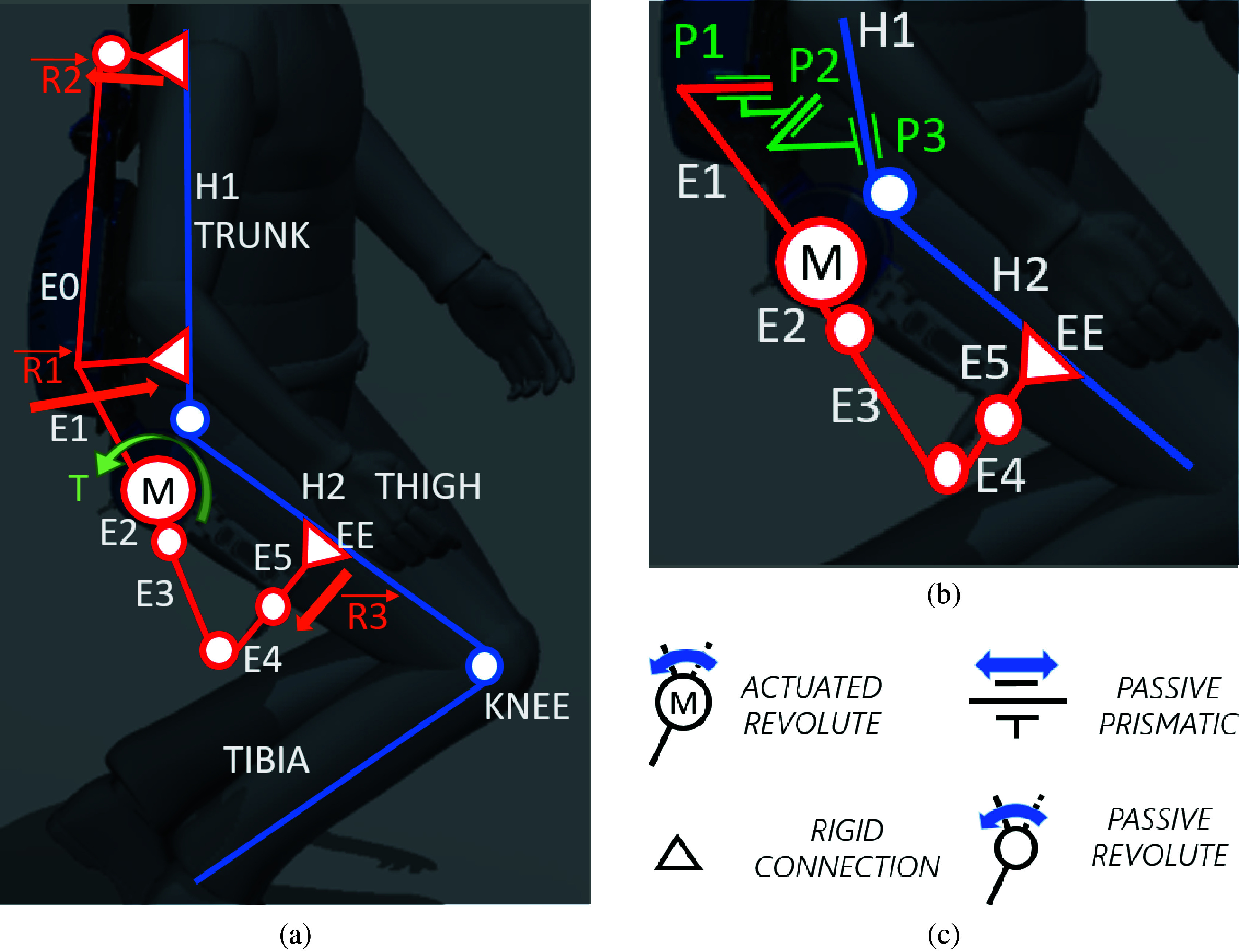
Sagittal view of the device on a mannequin in different positions. (a) The whole exoskeleton with the whole figure of the human body. (b) The presented model for simulation. (c) The symbols used in the previous figures.

XoTrunk, an upgrade to the design presented in Toxiri et al. (2015), is an occupational back-support exoskeleton designed to support manual material handling activities, reducing the risk of injuries at the lower back. This device is used in this work for the kinematic simulation and assessment. The model is constructed only for the right side, for sake of simplicity. The exoskeleton’s kinematic chain E is composed of a rigid frame, link E0 in Figure 3a, attached to user’s torso, link H1, using shoulder straps and a waist belt. Two motors, on the sides, are attached to the frame (revolute joint M between link H1 and link H2). They rotate the exoskeleton’s E3, E4, and E5 links that are connected via a garment (leg strap) to the user’s thigh H2, through a mechanical attachment, HT. The sequence of joints 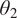 , 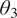 , 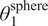 , 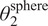 , 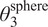 , and links E2, E3, and E4 creates an R–R–S (revolute–revolute–spherical) alignment mechanism to compensate for the motor’s ICR migration with respect to the hip ICR, like a standard passive R–R–R (revolute–revolute–revolute) compensation in Näf et al. (2019). In addition, these mechanisms are intended to fit the device to different body shapes and dimensions. The device is not rigidly connected to the user’s kinematic chain H (composed by links H1 and H2): straps, belts, and webbings, composing the braces, are compliant elements. Placement and stiffness of the materials have been chosen, with great focus on users’ comfort, to distribute reaction forces created during the exoskeleton’s assistive phase, to avoid slippage and thermal comfort. These physical attachments add an additional source of misalignment compensation (Näf et al., 2019).

The device can be operated in a “transparent” mode to follow the user’s movements without hindering walking or when no assistance is required. An on-board embedded computer analyzes the user’s posture and muscle activation (surface EMG collected with a Myo armband [North Inc., ON, CA]) to effectively provide an assistive motor torque *T* at the hip joint as described in Toxiri et al. (2018). The assistive torque T results in reaction forces R1, R2, and R3 on attachments in the Sagittal plane, in Figure 3a.

Waist belt and leg straps are analyzed in this work and to validate the method presented in this paper (model in Figure 3b). Leg straps can not unload on the body or absorb the parasitic reaction forces and torques created by Boundary or Internal singularities and poor fit. This results in the migration of attachment points. The waist belt noticeably migrates upwards during squatting and stooping. To account for this phenomenon in the model, virtual prismatic joints P1, P2, and P3 are added to the kinematic chain for the MIL simulation. These three joints introduce in the model both waist belt migration and fitting issues. In fact, the device has weak fitting performances for different body sizes and proportions, because only E2 and E3 chains provide for an adapting mechanism for different body sizes. The effectiveness analysis of the shoulder braces is out of the scope of this contribution, therefore, the model is composed only by the lower part of the exoskeleton and the human hip and leg. This work presents only a kinematic model, the kinetic simulation is out of the scope of this contribution.

### 2.2 Algorithm

**Figure 4.**
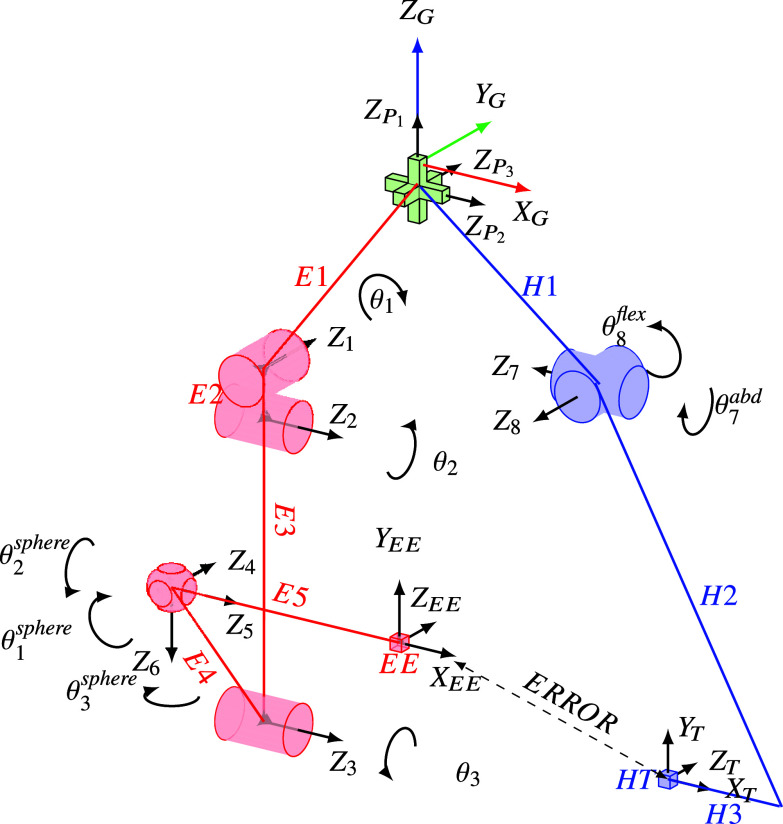
Kinematic diagram of the model for the presented simulation. In red exoskeleton chain E, green perturbation joints P and blue the lower limb chain H. Dashed line connecting exoskeleton’s EE and leg’s attachment point HT is the trajectory following Error used as one of the indexes for computing the exoskeleton’s performance.

XoTrunk’s kinematic chain E, is composed by six links, one actuated revolute joint and five passive revolute joints, as depicted in Figure 4. One end of link 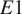 is connected to the world reference frame 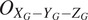 and is coincident to a part of the main exoskeleton’s frame, as depicted in Figure 3b. The actuated revolute joint is represented by the joint variable 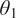 , while the passive joints are indicated by their joint variables 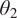 , 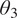 , 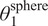 , 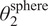 , and 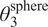 . Link E2 connects 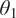 and 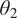 , link E3 connects 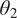 and 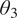 , link E4 connects 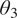 to the spherical joint, that is, composed of three coincident joints 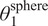 , 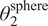 , and 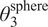 . Link E5 connects the spherical joint to the tip EE. Each joint of XoTrunk has a determined maximum RoM, imposed by shapes, dimensions, and safety. Whenever a joint reaches its RoM’s limits, saturation of the joint occurs. The simplified lower limb kinematic chain H in Figure 4 is composed of two links and two revolute joints. Hip flexion/extension and abduction/adduction are model by the two revolute joints 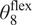 and 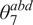 , respectively. Knee motion is ignored and the end point of link 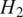 is free to move. The frame 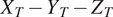 , named 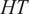 , represents the anchor point of the leg strap on the rear thigh. This is also the origin of the trajectory that the exoskeleton’s point EE is required to follow. The two kinematic chains are connected through the additional kinematic chain P, which consists of three coincident prismatic joints 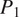 , 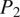 , and 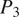 . The scope of this imaginary kinematic chain P is to simulate fitting offsets and waist belt migration during motion.

EW and LW are computed from RoMs; exoskeleton’s joints RoM are imposed by design while hip flexion/extension and abduction/adduction RoMs and lower limb dimensions are derived from data in tables from Pheasant (2003) and Boyer et al. (2012). In addition, Boyer et al. (2012) suggest that for unconstrained movements, the 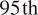 percentile of joints RoM should be considered. In Pheasant (2003) there are corrections for the added width of wearing work clothes. In this work, 1 cm is added to hip-width and thigh thickness. Input body dimension for simulations is reported in Table 1.

**Table 1.** Data derived from 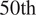 percentile of anthropometric estimates for British adult workers aged 19–65 years (Pheasant, 2003).

Gender	Hip width (mm)	Femur length (mm)	Thigh thickness (mm)	Thigh fixation pos. (mm)	Extension–flexion 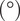	Abduction–adduction 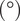
Male	360	375	160	175	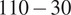	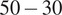
Female	370	310	155	110	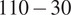	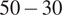

**Table 2.** Denavit–Hartenberg parameters for E chain in Figure 4.

Link	*a*	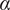	*d*	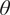
E1	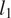	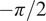	0	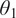
E2	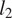	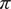	0	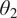
E3	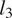	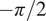	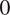	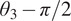
E4	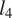	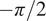	0	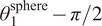
E4	0	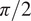	0	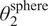
E4	0	0	0	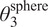
E5	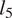	0	0	0

**Table 3.** Denavit–Hartenberg parameters for P chain in Figure 4.

Link	*a*	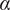	*d*	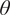
1	0	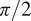	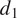	0
2	0	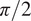	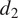	0
3	0	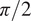	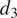	0

**Table 4.** Denavit–Hartenberg parameters for H chain in Figure 4.

Link	*a*	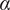	*d*	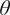
1	0	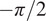	*hw*/2	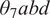
2	0	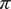	0	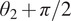
3	−*fl*	0	–*tt*/2	0

Abbreviations: *fl*, femur length; *hw*, for hip-width; *tt*, thigh thickness.

**Figure 5.**
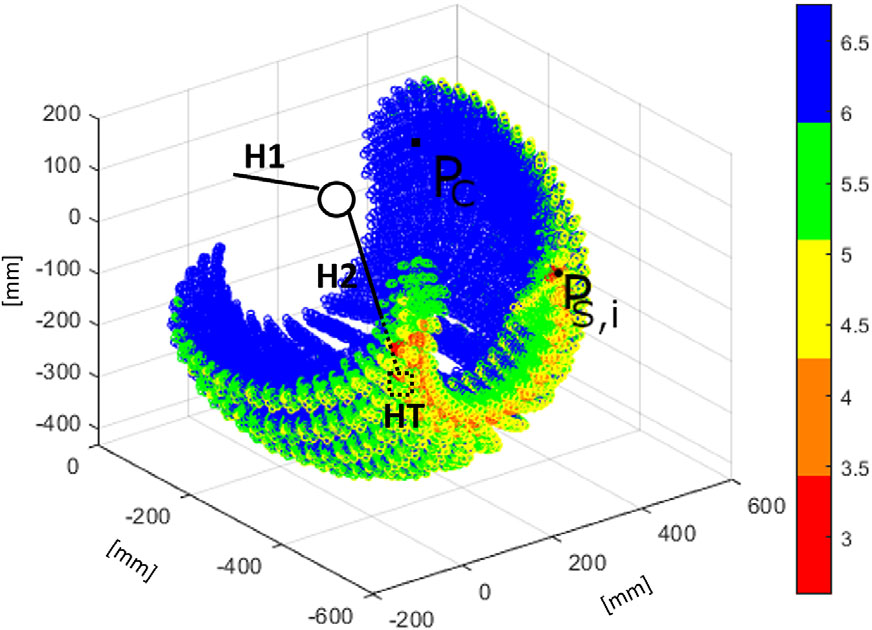
Manipulability index 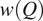 obtained from the workspace simulation of the XoTrunk kinematic chain. The color scale is logarithmic, red points are those closest to singularity points. The figure shows that only a little volume of the exoskeleton’s EE workspace is near a singularity point. The area with singularity points is not overlapping with the leg’s attachment point HT workspace.

When linked together, E, H, and P create a closed loop. This closed-loop chain needs to be analyzed in order to avoid singularity points within the computed workspaces (Gosselin and Angeles, 1990). Singularity points should not be included in the EW as no IK solution can be obtained for those points. Zacharias et al. (2007) describe a method to avoid the inclusion of false-positive points in the workspace created only by DK: manipulabilty analysis. Manipulability is defined as a quantitative measure of a robot’s ability to change the position and orientation of its EE tip (Yoshikawa, 1985). When the manipulability value is 0 or close to 0 the robot reached a singular configuration in its state space variables. This results in a local dexterity reduction or complete inability to move. Creating workspaces through DK is faster than using IK (i.e., evaluating the Cartesian sampled points 
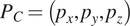
 in the Cartesian space, depicted in Figure 5) or an hybrid DK + IK solution (Porges et al., 2015). However, creating a workspace using only DK does not account for singularities points in the joint space 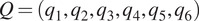 as IK does, hence creating false positive within the simulations. Therefore, singular joint space points 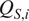 collapse into the same workspace point 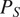 (in Figure 5). Even the neighboring joint space points of 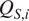 are mapped into points close to 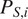 . Therefore creating a densely populated workspace area that should not be considered valid for any calculation.

The algorithm to evaluate the trajectory tracking performance of the exoskeleton EE tip, take into account the exoskeleton’s and human’s kinematic chain divided. In this manner, the singularity points of E and H can be evaluated separately. However, the manipulability index 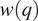 is calculated for the exoskeleton model only. Leg singularities configurations can only arise at the limits of joints 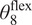 and 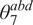 mobility (Boundary Singularities). The manipulability index 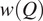 is state dependent and, for every 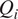 configuration, the Jacobian needs to be computed. For a redundant kinematic chain, 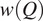 is calculated using Equation (1) (Yoshikawa, 1985).
(1)

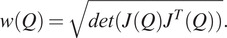



Applying Equation (1) to EW results in the representation shown in Figure 5. This figure shows that the exoskeleton’s singularities lie on the workspace borders, only. The borders’ area will not be used in any of the calculations because LW does not overlap those areas. Therefore, we can create EW with DK, resulting in faster computation without any false positive points in the analysis.

**Table 5.** Percentage of primary workspace points belonging to each value range in the color bar.

Value	Points %
6+	50.2
5–6	37.8
4.2–5	10.2
3.5–4.2	0.6
0–3.5	1.2

*Note.* More than 85% of the whole workspace’s points are far from singularity points.

**Figure 6.**
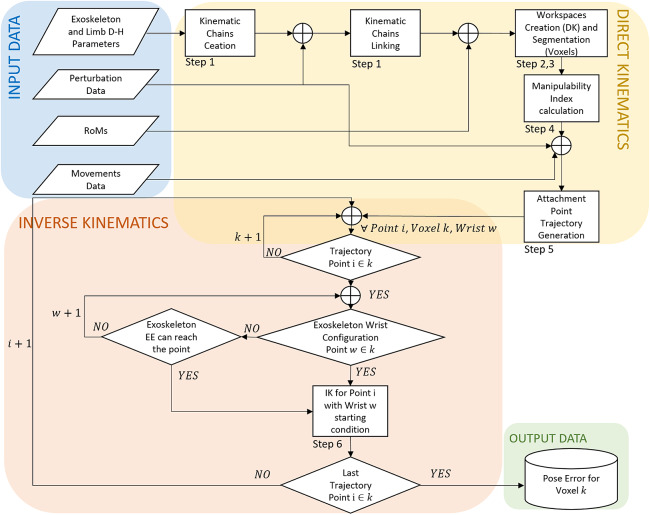
Flow chart representation of the described method. Input and output data are stored in Matlab *.mat* files. The figure shows also the step numbers as described in the section “Algorithm.”

The presented framework is developed as a collection of Matlab (The Mathworks, Natick, MA) functions and uses of the standard *Robotics Toolbox*. Mathematical description of the tools used is reported in Appendix A. The whole process is presented in Figure 6 and can be summarized in the following steps:

1. ***Kinematic Chain Creation:*** firstly, the software is provided with Denavit–Hartenberg parameters for the human and the exoskeleton’s kinematic chains. This step uses *RigidBody Tree* objects provided by the *Robotics Toolbox*. In this step, the exoskeleton, limb, and perturbation chains are linked together.
2. ***Workspaces DK Computation:*** in this step, only the DK algorithm is used to calculate the Cartesian workspace from a sampling of the E, H, and P state space, obtaining the points *w*. State space is sampled with a uniform distribution spaced sampling. The exoskeleton’s workspace calculation is limited to the first three joints (motor and passive revolute), thus defining a *Primary Workspace* (Gupta, 1986). In fact, the last three revolute joints assembled as a spherical joint have a small workspace, that is independent of other joints configurations. The evolution of Primary Workspace is used to calculate the Reachability Index. In addition, neglecting the spherical joint movements (i.e., the spherical joint is fixed) drastically reduces the required computational time from 414 to 30.5 s (under same conditions).
3. ***Workspaces Discretization:*** the segmentation of the workspace into voxels is performed only over the Cartesian space where both the exoskeleton’s and user’s limb can overlap. Voxels are visual and logical entities, they do not represent a boundary or constraint to leg or exoskeleton movements. Voxels simply partition the Cartesian space and hold reference of all Primary workspaces points, the state space configurations that create the workspace points, Reachability and Capability index values and IK solutions. In this way, it is possible to stop and resume simulations and the time consumption of IK algorithm can be optimized. Voxel dimensions can be varied; bigger voxels require less time for data accessing but can be misleading during evaluation steps. Lowering the volume threshold can lead to the condition that every Cartesian point in the voxel is out of EE workspace for every primary workspace’s point *w* in the voxel. A voxel size of 2 cm length per side, is used.
4. ***Manipulability Index Calculation:*** The manipulability index is calculated for every point *w* in the primary EW, computed in Step 2. This avoids to include false-positive workspace points 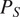 while computing the IK or evaluating *Reachability* (presented in the section “Reachability”), Primary Workspace, and *Capability* (presented in the section “Capability”). The IK solver will take as its initial configuration a point *w*, which its joint space coordinates that must be far from Internal Singularities.
5. ***Attachment Point Trajectory Generation:*** joint space trajectories are imported from data collected from real subjects during experimental assessment using the protocol described in the section “Experimental design and protocol.” Cartesian space trajectories of the leg attachment HT are computed once for each subject, and voxels containing them are marked valid for IK solution calculation. Perturbation trajectories, from joint P1, P2, and P3, are added afterward. In fact, the virtual prismatic joints are oriented with the world reference frame so that a pure translation can be added.
6. ***Trajectory Following Error IK Calculation:*** this iterative step takes place for every trajectory point *i* of attachment HT in Cartesian space. Firstly, the trajectory is built with DK imposing the recorded values on 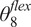 and 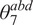 . Then, the algorithm checks if every point *i* belongs to a voxel *k* that contains both primary EW points *w* and leg ones, otherwise point *i* is discarded. Indeed, if a trajectory point created by HT lies out of the LW it is considered a calculation error or an error in the recorded hip joint angles. If point *i* lies in adjacent voxels within reach of the exoskeleton’s EE, the IK algorithm will be executed anyway. An *ik* object, from the Matlab *Robotic Toolbox*, allows us to set different constraints for the calculation of IK solutions. In this work *Pose Target* and *Joint Boundaries* constraints are used, HT and EE are associated by their pose in Cartesian space (i.e., position and orientation with respect to an origin). This configuration ensures continuity in joints space between solutions, setting an initial estimate for the joints space and setting the maximum allowable position and angle error. If the numerical solver does not converge to a solution within a *Maximum Time* and *Maximum Random Restart* iterations, then it delivers the best available solution. The solution provides joints configuration and the relative pose error.

### 2.3 Reachability

Reachability, firstly introduced by Guan and Yokoi (2006), is an index that measures the ability of kinematic chain to reach a certain point in the Cartesian space. Zacharias et al. (2007) extended the definition to include preferred directional paths for anthropomorphic robotic arms. In this work, Reachability index 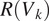 is defined for exoskeletons as the fraction of LW points *l* that are distant link’s 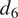 length from a point *w* of the primary EW. A reachability index is associated to a voxel 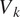 and is the average value of the reachability of all points *w* in 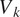 . Reachability index 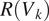 has ranged from 0 to 100%. If 
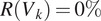
 means that no points *l* are reachable from any point *w* in 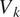 , a value 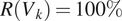 means that all the points *l* are reachable from all points *w* in 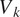 . Reachability index is influenced by the users’ anthropometry, exoskeleton’s fit, and the length of link 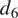 . If the exoskeleton is improperly worn the compensation mechanism will not work in its desired condition, resulting in low reachability. Increasing links length will improve reachability but this solution will impact negatively on users’ comfort as the exoskeleton’s encumbrance will rise as well. Given 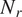 and 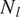 the cardinality of the set of the reachable LW and the cardinality of LW in 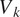 , 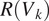 is calculated as follows
(2)

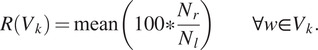



**Figure 7.**
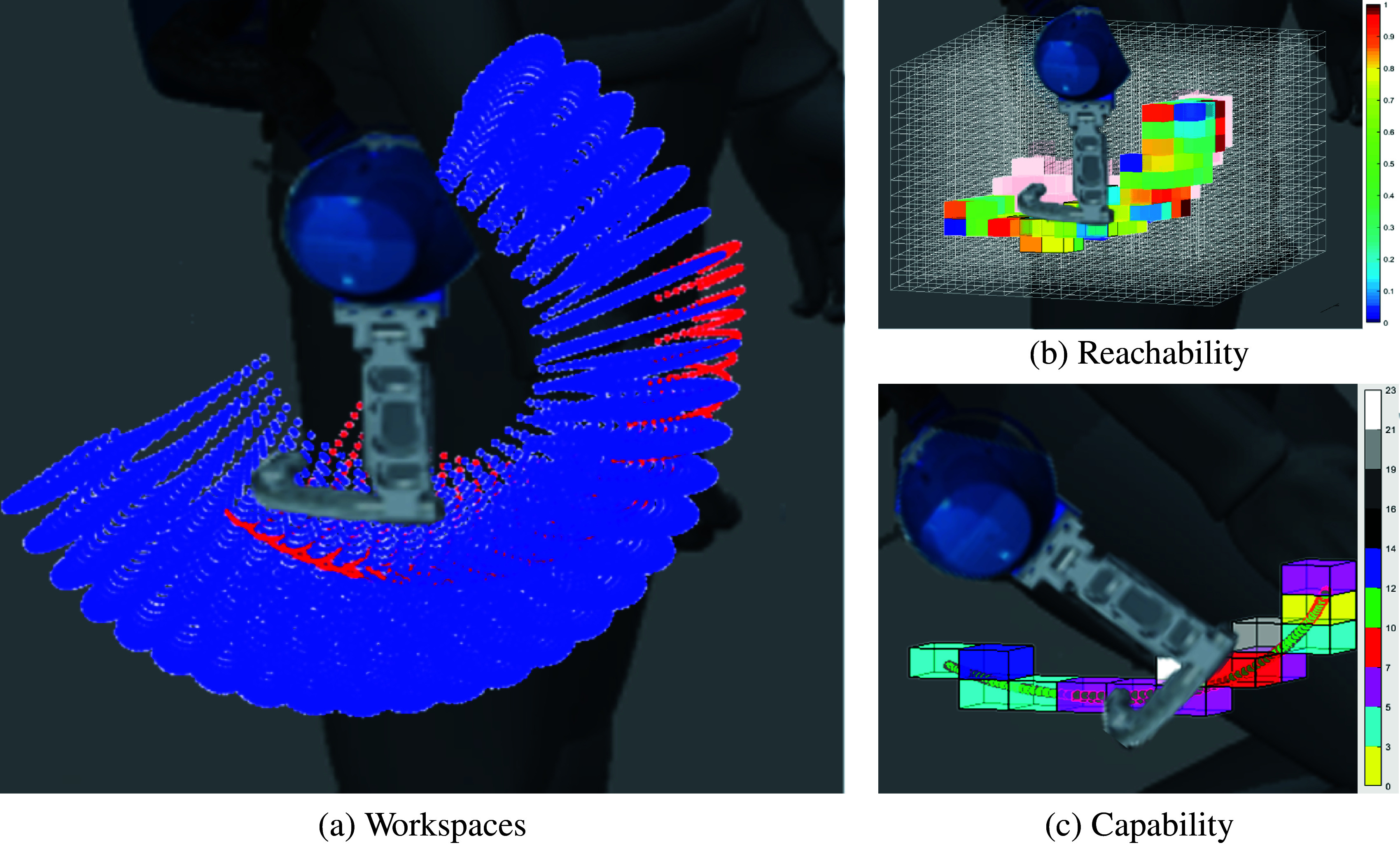
Visual representation of simulated data for a male subject (anthropometric data in Table 1). In (a) there is the primary EW (Dextereous fraction) in blue dots, while the primary LW created by HT is depicted in red dots. Light red voxels in (b) represent the space where the primary EW is not present. Higher values of Reachability means that the exoskeleton’s 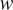 points can reach more of the leg’s 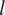 points in the same voxel, resulting In (c) green dots are simulated trajectory points that EE is imposed to follow.

The index defined as in Equation (2) allows us to visualize the changes of the primary EW vs. different initial belt’s position caused by different fittings and body sizes. Figure 7a is provided as an example of 3D spatial representation of workspaces. Calculation is based on a virtual male mannequin, with data from Table 1, wearing XoTrunk. Blue dots represent EW while red ones represent LW. Using this representation, however, it is unclear if the exoskeleton fully covers the LW with this particular fit on this particular model. To have a clear view on the intersection of the Primary workspaces it would require an interactive image to roll or several images with a different point of view. Figure 7b shows the same example representing a Reachability map to overcome the problem of a classical workspace graph. With respect to Figure 7a,b can show immediately if the two workspaces overlap, and where the LW do not overlap, light red voxels area. This means that the fit of that exoskeleton in this example is not optimal to cover all of the possible leg’s movements. Because Reachability maps are computed over the whole workspaces, they are not fully informative on the exoskeleton’s performance in assisting users in different tasks. Occupational exoskeletons are designed to assist certain tasks, that means that a back support exoskeleton should assist the users while lifting, lowering, or carrying loads. Those tasks do not require point HT to move over its whole workspace but to move along trajectories in its workspace, that reachability can not display. Reachability index is useful to optimize the shape of EW, given a desired body attachment’s (e.g., point HT) workspace to cover. Indeed, Figure 7b shows areas where there is overlap in solid colors, that correspond to the workspace created by hip flexion, extension, and abduction. The workspace created by hip adduction is not fully covered but it is not a movement performed during lifting, lowering, or carrying loads (e.g., the leg do not cross). However, Figure 7a shows that a notable part of the primary EW is never overlapped by HT workspace; this could suggest a rearrangement of the passive DOFs.

### 2.4 Capability

Capability was first introduced by Zacharias et al. (2007) and its definition expanded by Porges et al. (2015). Their definition provides a functional evaluation of the dexterity of a mobile anthropomorphic robotic arm. The *Capability* map is defined as a compact representation of dexterity evaluated over a directional structure, in the Cartesian space. In this work, we define an exoskeleton’s Capability as the quality measure of the exoskeleton’s dexterity: its ability to accomplish desired task without transmitting parasitic forces unloading on the braces. The directional structures, on which to evaluate Capability, are the trajectories of HT; those trajectories are computed from user’s movements. In order to achieve a compact and visual representation, Capability 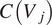 has to be associated to any voxel in EW, that is, relevant for capability (i.e., contains at least one HT trajectory point). Any other voxel, discretizing the EW in Cartesian space, is not considered in the calculations. Considering the type of exoskeleton used for simulation and the kind of assistance delivered, Capability is therefore defined as follows: given the terminal point of the exoskeleton EE, 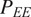 , and the terminal point of the leg attachment HT, 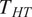 , Capability is proportional to the pose following error 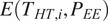 . Since Capability is a local quality measure, a voxel 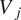 Capability is the inverse of the average maximum error value computed for the exoskeleton space state configurations 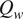 and the trajectory points 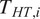 that belong to that voxel.
(3)

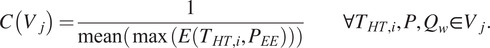



Capability index is an adimensional scalar value, its lower threshold is zero and has no upper threshold (i.e., a zero value error in pose following will result in an infinite Capability index). High Capability values in a voxel 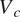 mean that for every 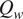 and 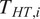 in 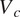 the average pose following error is little. Therefore, there will be little deformations of the textile garments and the limb’s soft tissues imposed by the pose error. This situation will result in no discomfort to the user that will not work against the exoskeleton to complete the movement. Figure 7c shows in Cartesian workspace the trajectory points created by HT, in green dots, and the voxels containing the trajectory with their colorbar to interpret the values. All the other voxels are transparent, they do not contain any trajectory points and no solution to the IK exists.

## 3 Experimental Validation

“Methods” section describes the kinematic structure of the interacting bodies (i.e., back-support exoskeleton and human body) based on assumptions and goals outlined in the section “Back-support exoskeleton.” In “Results” section, we validate the preliminary model using a small cohort of test subjects, to show the potentialities of the proposed framework. Hip joint movements, exoskeleton’s motor angular position, and the mutual position of leg and exoskeleton origin frame are collected to validate the simplified kinematic model developed for the simulations. In this section, we present the experimental setup and protocol.

### 3.1 Physical Setup

**Figure 8.**
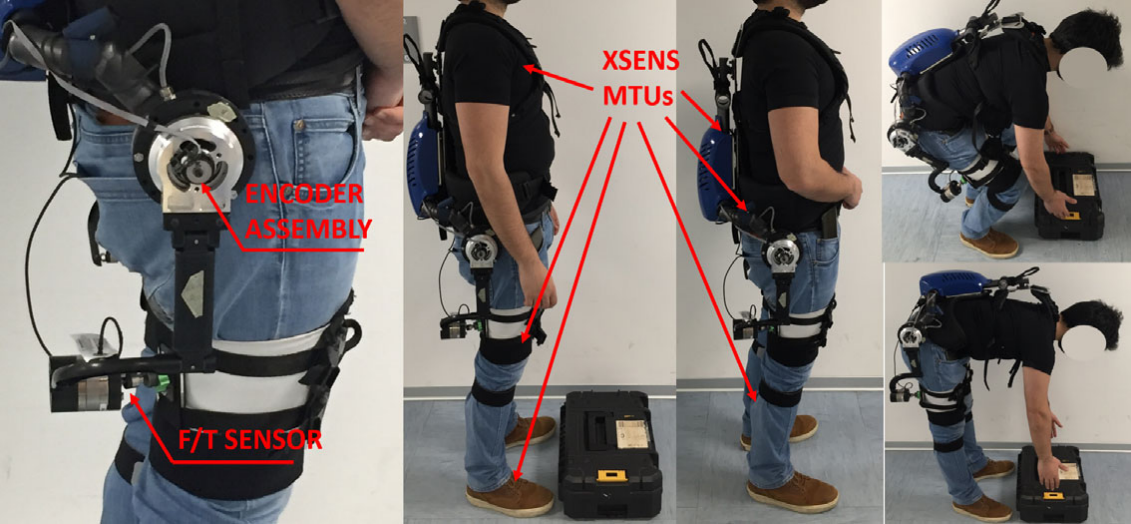
Physical setup of the preliminary assessment. The Xsens MTws IMU is secured with proprietary hook and loop wraps and t-shirt. On the left, zoom visual of the instrumented exoskeleton with encoder assembly and the force-torque sensor on the end of the exoskeleton’s link E4. D-H parameters are changed to consider the F/T sensor dimensions.

**Figure 9.**
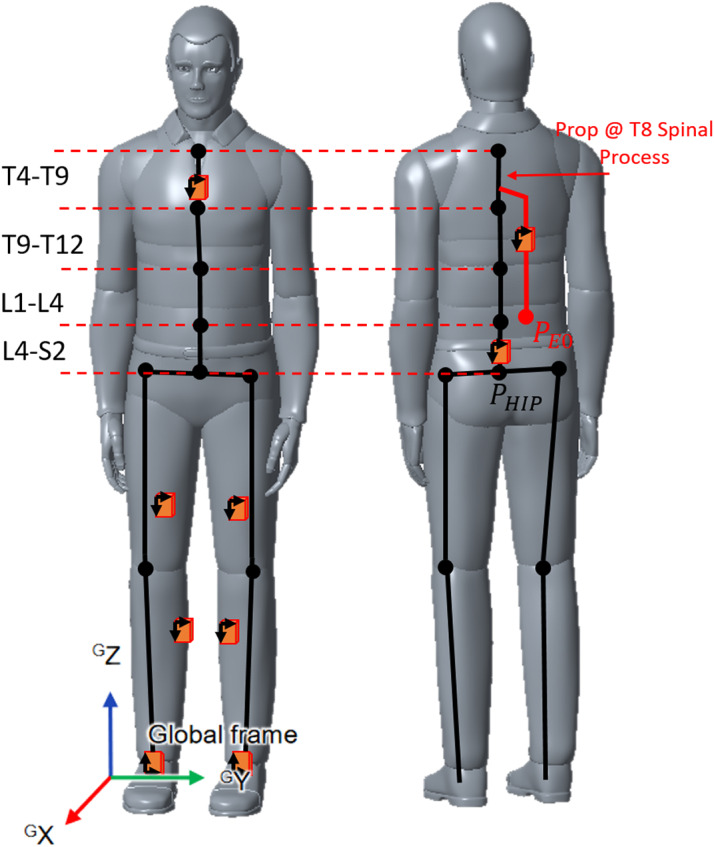
Placement of MTUs on human body in the *lower body + sternum* configuration available in Xsens acquisition software. The black skeleton shows all the joints and links computed by the Fusion engine by default, the red links and joint represent the custom Prop added to compute exoskeleton and waist anchor point drift in time.

For data recording, a “sensorized” XoTrunk copy was used. This modified exoskeleton has no actuators but has additional sensing capabilities including absolute encoder assemblies at the 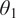 joints and a Mini58 F/T sensor (ATI Industrial Automation, Apex, NC) placed at the end of exoskeleton’s link E4, Figure 8. To record the subject’s motion data, an XSens MTW Awinda (Xsens Technologies B.V., Enschede, The Netherlands) is used. This sensor system is composed by several wireless Motion Trackers (MTw). These MTws are inertial sensors that can be worn under the exoskeleton and no mechanical interferences were recorded during donning and doffing of the two systems on the test subjects. The system is set up to record torso and lower body movements. Figures 8 and 9 show the placement of MTw inertial sensor units (IMU) on the subject’s body. XSens is also used to record the relative motion of the leg and exoskeleton kinematic chain origin frames. Unlike a camera motion tracking system, the XSens does not suffer from marker’s occlusions.

Xsens MVN Studio software contstructs a digital skeleton of the users (implementing an LSM), this representation is based on their anthropometric data and shows the users' movement real-time. MVN Studio allows to add an additional segment to any point of the LSM, the Prop segment. To calculate the exoskeleton’s origin frame drift with respect to the leg chain origin frame, a custom Prop segment is added to the LSM like in Figure 9, and an additional MTw is fixed to the exoskeleton. To recreate movements in the Cartesian space, the XSens Fusion engine uses quaternions for each body segment and additional velocities and accelerations to compensate for drift in the IMUs. The global origin 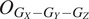 reference frame is set to be coincident with the subject’s right heel. Each body segment is associated with a body reference frame *B*. Physical dimensions in *B* and a quaternion are enough to represent a rotation with respect to the world frame *W*. To reconstruct the exoskeleton’s origin frame movements, the same approach was used. The Prop is attached to the position of T8 spinal process (available from Xsens) and is given the dimension of E0 link, in Figure 3a. In this way, it is possible to compute the movement of the exoskeleton’s belt as the geometric distance, 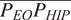 , between points 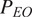 and 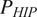 in Figure 9. 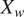 and 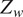 values are the coordinates on the origin coordinate frame 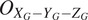 of 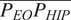 . The Xsens Fusion Engine outputs all the dimensions and movements in Cartesian space and quaternions needed to compute any point movement. An explanation of the reconstruction of the movements using quaternions is reported in Appendix B.

### 3.2 Experimental Design and Protocol

Three healthy male volunteers (age 29.6 (1,3) years, height 180.0 (2) cm) participated in the trials. The experiments are compliant with the experimental protocol approved by the Ethical Committee of Liguria (protocol number: 001/2019). Each subject was instructed to repeat lifting and lowering movements at a natural self-determined speed while varying the *lifting technique* (squat or stoop). Each trial run consisted of five repetitions. Each subject was asked to perform three runs for each lifting technique.

**Figure 10.**
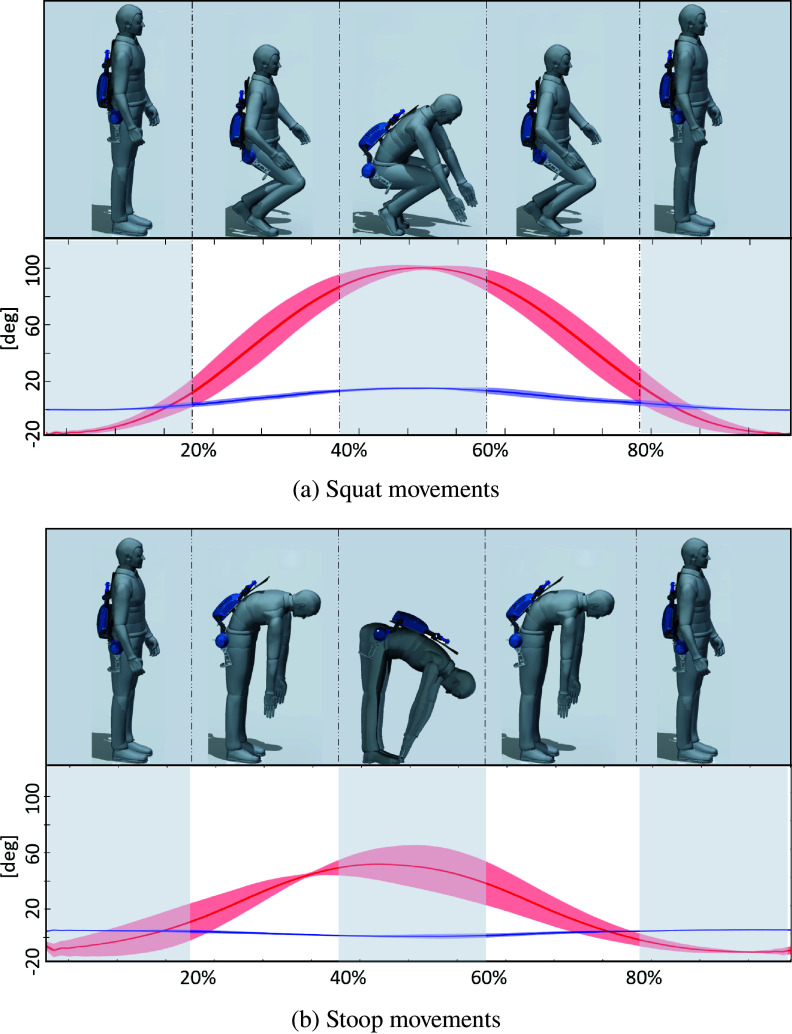
Graphs of the movements performed during validation experiments. Hip flexion and abduction angles (depicted in red and blue, respectively) are segmented to show the overall movement performed. Solid line represents the average value of the angles, shade represents standard deviation across all the recorded movements.

Anthropometric data for the MIL simulation were collected directly for each subject (thigh circumference, leg attachment position) or computed from the Xsens Actor data configuration (thigh length, crotch width). To perform squats the subject was asked to stand up in a natural position while wearing the instrumented exoskeleton, shown in Figure 10a from 0 to 20% of the movement. Then the subject was instructed to bend to reach a box placed at his feet (Figure 8), also the knee bending was permitted in this lifting condition. To perform a stoop, Figure 10b, the subject is asked to reach the box at his feet without bending his knees. Shoulder, waist, and leg straps were visually checked at every repetition to ensure that these braces were always secure.

**Table 6.** Anthropometric data of the three subjects.

Subject	Height (cm)	Body rise (cm)	Hip width (cm)	Thigh width (cm)	Thigh length (cm)
S1	180	49	32	17	45
S2	177	48	35	17.5	47
S3	181	46	40	18.5	51

From the instrumented exoskeleton and Xsens Awinda, we extract and compute *body kinematics, exoskeleton actuator angles and attachment points drift*. These data are necessary to perform the MIL simulation, which formed the second step for the experimental evaluation. A different simulation is run for each combination of body kinematics and lifting techniques for a total of 18 different simulations. Every simulation is computed with the same *ik* object parameters (as presented in the section “Algorithm”), voxels dimension, workspace sampling rate, and type (i.e., uniform sampling).

### 3.3 Data analysis

To show a qualitative correlation between exoskeleton’s abilities and parasitic forces creation at the braces, we need to collect experimental data to feed as input variables to the simulations from Xsens and motor encoder. A quantitative assessment would require an extension of the model to be able to compare predicted forces and torques and the recorded ones. This is out of the scope of this paper. Due to the orientation of the F/T sensor with respect to the assistive reaction forces (Figure 3a) relevant recorded values, are :

- *X and Y axis Forces Norm*
- *X and Y axis Torques*

They represent any parasitic force and torque, that is generated by the exoskeleton and unloaded on the user’s limb. The IK solutions are computed from the simulations in the second step of the validation. Relevant values from the solutions are:

- *norm of the pose error*
- *joint configuration solution*
- *E and H workspaces points*

**Figure 11.**
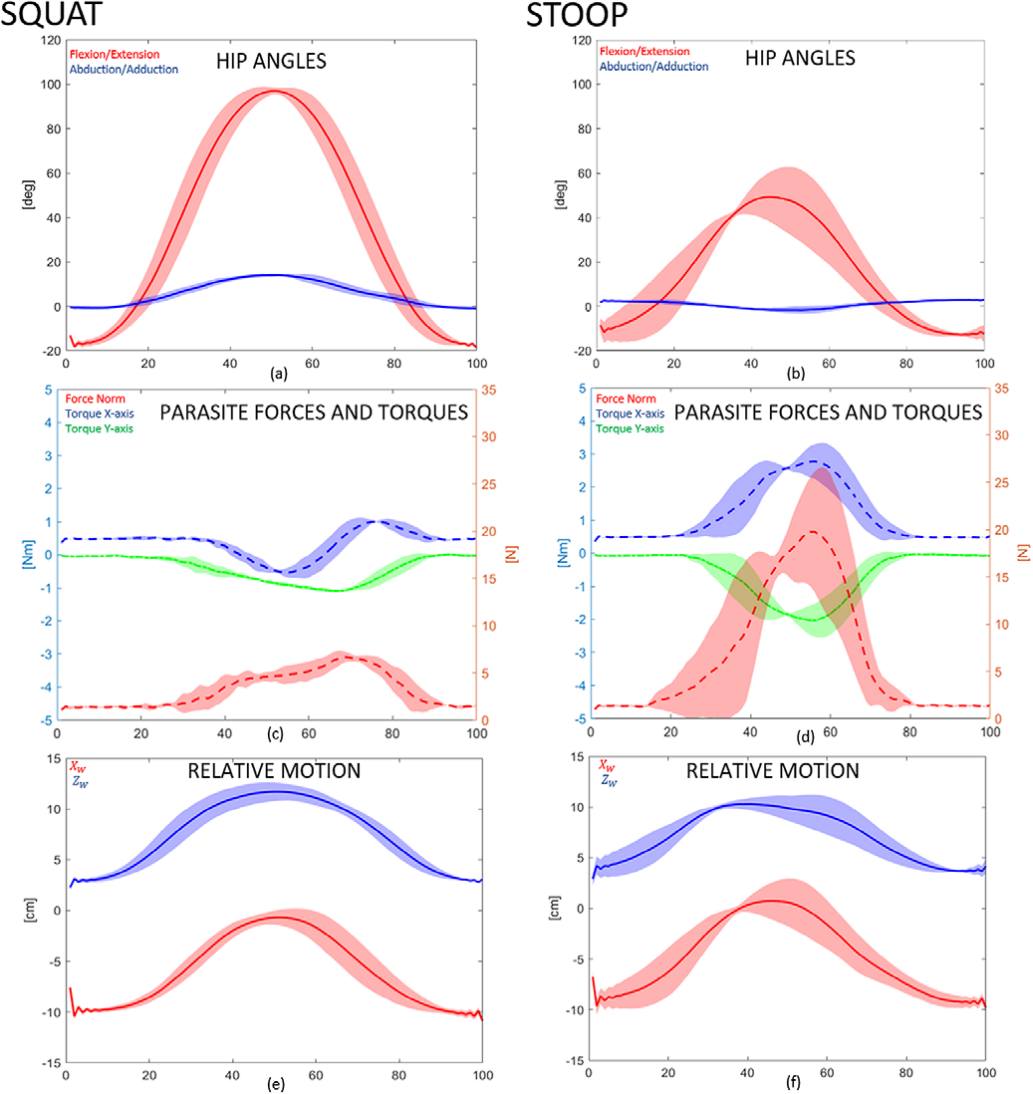
First test subject’s data recorded for validation. Data plotted is the mean value (dashed line) and standard deviation (fill) of data recorded and simulated for all three runs for different lifting techniques, from 0 to 100% of the movement cycle. Figure 11a,b shows hip joint angular motion. Figure 11c,d shows recorded forces and torques, while Figure 11e,f shows 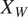 and 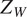 .

**Figure 12.**
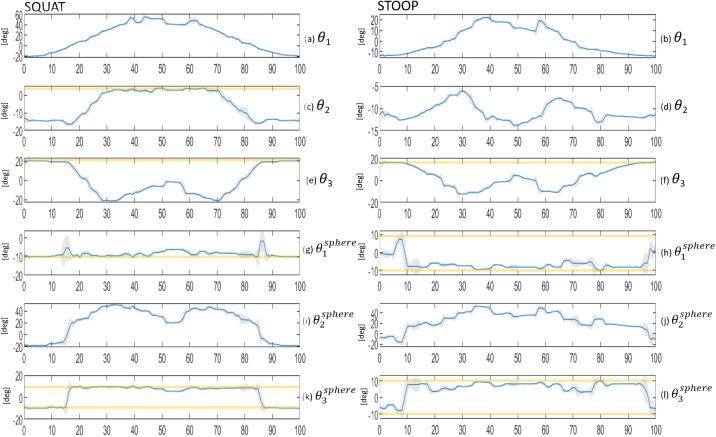
Exoskeleton’s joint angles were simulated for validation with S1 movements’ data input. All figures show angular values of 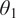 to 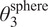 joints’ variables, from 0 to 100% of the performed movement. All the simulated joint variables of the exoskeleton are present. Figure 11g–l shows the joint variables of the spherical joint before the EE tip. Orange bars show the saturation threshold (i.e., end of ROM) only on for the variables that have saturated in the simulations.

**Figure 13.**
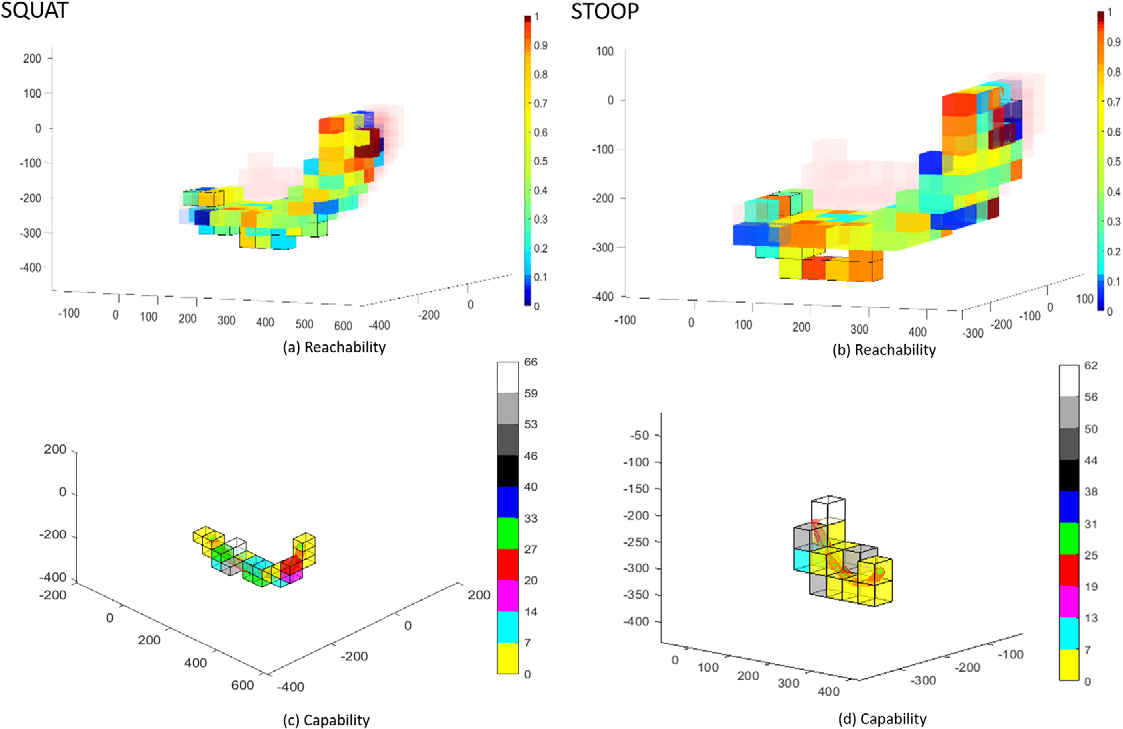
S1 subject’s data simulated for validation summarized in Reachability and Capability maps. Left side squat movement, right side stoop. Capability maps in (c and d) are reported with the same point of view with respect to Reachability maps in (a and b). The voxels in the Capability maps are a subset of the voxels shown in Reachability maps. The color bars show normalized values in (a and b) and maximum values in (c and d).

**Figure 14.**
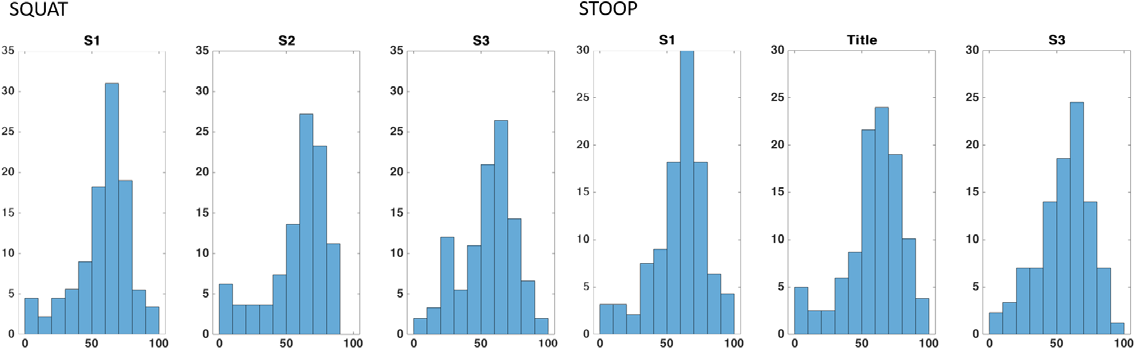
Percentages of the total voxels 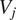 within a determined interval of Reachability values. The intervals stop at 100, that is, the maximum Reachability value by definition. The values reported here are averaged on the total of 15 repetitions (divided into three runs) of each movement.

**Figure 15.**
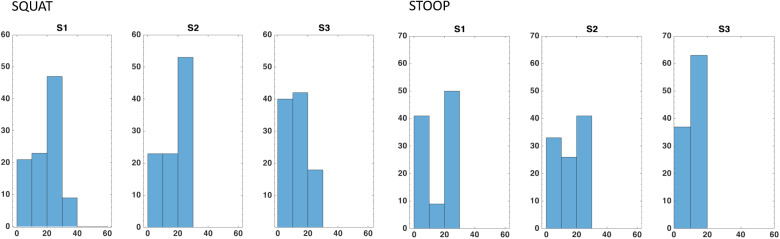
Percentages of the total voxels 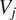 within a determined interval of Capability values. The intervals stop at 60, that is, the maximum Capability value obtained in a single repetition. The values reported here are averaged on the total of 15 repetitions (divided into three runs) of each movement.

Data from IK solutions, Xsens, and instrumented exoskeleton were averaged across the five repetitions for any of the relevant value to mitigate any possible movement execution errors. Because no execution speed was imposed on the subjects and the sensors have a different sampling rate, recorded data were segmented using hip flexion repetitive peaks and resampled to have a matching length. The common sampling rate used is 60 Hz. The results are presented from 0 to 100% of the trial movement cycle, for consistency. To qualitatively evaluate the model’s correlation with the measured forces and torques, EW points in each voxel are selected with the following criterion: a workspace point 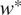 is acceptable as starting condition for IK calculation only if 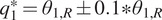 , where 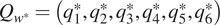 is the state space configuration of 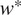 , 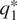 , and 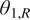 are respectively, the simulated and recorded motor joint angles. The experimental results are reported separately for the *squatting* and *stooping* lifting techniques. In Figures 11–13 data for subject S1 are reported. Each plot in Figures 11 and 12 shows average values as a line and standard deviation across all repetitions and runs for S1 subject. Left column shows squatting movement, while the right shows stooping. Tables 7–10 and Figures 14 and 15 report data for all movements and all subjects (S1, S2, and S3).

**Table 7.** Direct and indirect anthropometric measures regarding all subjects and all movements.

Subject	Movement	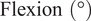	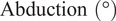	Drift 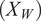 (cm)	Drift 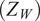 (cm)
S1	Squat	[**−18.5**(1), **97.7**(2.2)]	[**−1**(0.6), **14.2**(2.2)]	[**−10.8**(0.004), **0.5**(0.007)]	[**2.3**(0.001), **11.7**(0.01)]
S2	Squat	[**5.6**(12.9), **92.5**(12.5)]	[**−0.3**(2), **12.2**(2.2)]	[**−12.6**(0.6), **10**(1.6)]	[**2.5**(1.1), **9.3**(1.6)]
S3	Squat	[**−6.3**(6.3), **95.2**(18)]	[**−17**(0.9), **−1.6**(2.2)]	[**−10**(0.01), **1.9**(0.001)]	[**4.6**(0.01), **10.1**(0.01)]
S1	Stoop	[**−14.3**(0.7), **51.5**(9)]	[**−2**(1.1), **3**(0.3)]	[**−9.8**(0.006), **1.2**(0.01)]	[**2.8**(0.006), **10.5**(0.002)]
S2	Stoop	[**−3.7**(3.5), **88**(2.7)]	[**−17**(0.7), **5.2**(0.4)]	[**−12**(0.4), **−7**(0.5)]	[**1.8**(0.4), **7**(2.2)]
S3	Stoop	[**−14**(1), **76**(1.7)]	[**−18**(0.2), **−0.2**(1.2)]	[**−12**(0.001), **7**(0.01)]	[**3.5**(0.001), **9.6**(0.002)]

*Note.* In bold maximum and minimum values, in parentheses, standard deviation of the values averaged for repetitions and trials.

**Table 8.** Direct force and torque measures regarding all subjects and movements.

Subject	Movement	Force (N)	Torque 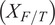 (Nm)	Torque 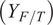 (Nm)
S1	Squat	[**0.9**(0.15), **6.8**(0.6)]	[**−0.6**(0.06), **1.9**(0.05)]	[**−1.1**(0.02), **0.01**(0.005)]
S2	Squat	[**0.8**(0.7), **3**(0.5)]	[**0.43**(0.04), **0.9**(0.3)]	[**−0.5**(0.3), **0.09**(0.04)]
S3	Squat	[**1.7**(0.5), **27.5**(4.3)]	[**0.4**(0.01), **3.2**(0.05)]	[**−3.5**(0.01), **0.01**(0.02)]
S1	Stoop	[**0.9**(0.3), **21.6**(0.6)]	[**0.4**(0.005), **3**(0.2)]	[**−2.24**(0.17), **0.01**(0.006)]
S2	Stoop	[**1.3**(0.07), **19.2**(3.8)]	[**0.43**(0.01), **2.8**(0.2)]	[**−2.2**(0.2), **−0.05**(0.01)]
S3	Stoop	[**1.2**(0.3), **32.3**(0.6)]	[**0.3**(0.02), **4.6**(0.04)]	[**−3.4**(0.03), **−0.05**(0.01)]

*Note.* In bold maximum and minimum values, in parentheses, standard deviation of the values averaged for repetitions and trials.

**Table 9.** Exoskeleton’s joint minimum and maximum values (in bold), averaged over all run and for every different subject and movement.

Subject	Movement	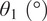	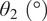	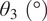	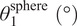	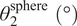	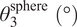
S1	Squat	[**−19**(0.9), **75**(2.3)]	[**−18**(0.4), **15**(0)]	[**−35**(3.8), **20**(0)]	[**−10**(0), **10**(0)]	[**−19**(1.7), **108**(42.5)]	[**−10**(0), **10**(0)]
S2	Squat	[**0**(0), **72**(1.7)]	[**−17**(3.6), **15**(0)]	[**−56**(3.3), **15**(8.4)]	[**−10**(0), **10**(0)]	[**−45**(43), **61**(5.4)]	[**−10**(0), **10**(0)]
S3	Squat	[**−4**(4), **73**(20.5)]	[**−43**(2), **1.7**(12)]	[**−33**(54), **3**(20)]	[**−10**(0), **10**(0)]	[**−87**(26), **154**(11)]	[**−10**(0), **10**(0)]
S1	Stoop	[**−17**(1.5), **29**(0.6)]	[**−20**(0.6), **0.4**(0.7)]	[**−28**(4.6), **20**(0)]	[**−10**(0), **10**(0)]	[**−52**(9), **61.2**(2.8)]	[**−10**(0), **10**(0)]
S2	Stoop	[**−3.9**(4.3), **81.5**(3.1)]	[**−24**(0.2), **10**(7.5)]	[**−49**(18.5), **20**(0)]	[**−10**(0), **10**(0)]	[**−51**(31), **55.4**(2)]	[**−10**(0), **10**(0)]
S3	Stoop	[**−9.8**(1.5), **36**(6.6)]	[**−41**(0.9), **−14**(1.4)]	[**−8.4**(1.5), **20**(0)]	[**−10**(0), **10**(0)]	[**−32**(11.4), **137**(15)]	[**−10**(0), **10**(0)]

*Note.* A value with zero variance can be interpreted that the joint reached saturation.

**Table 10.** Exoskeleton’s joint saturation percentage during the recorded movements (i.e., squatting and stooping) for all the subjects.

Subject	Movement	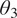 (%)	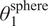 (%)	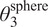 (%)
S1	Squat	26	63	24
S2	Squat	0	60	15
S3	Squat	3	91	23
S1	Stoop	7	57	4
S2	Stoop	8	67	13
S3	Stoop	39	80	14

## 4 Results

Figure 11a,b show hip flexion–extension angle and abduction–adduction data. For the squat movements, in Figure 10a, hip flexion can rise over 60° in combination with knee flexion, but for the stooping, Figure 10b, hip flexion never goes above 60° (Pheasant, 2003). Squatting results in hip abduction to allow the arm to reach the box placed on the floor in Figure 8. Conversely, stooping results in hip adduction. In fact, as seen in the interval between 20 and 40% of the movement cycle in Figure 10b, the abduction angle decreases below zero for all subjects (with a minimum of −17° for S3). The force vector norms and torques are presented in Figure 11c,d. Higher force norms are recorded during stooping, with a maximum increase of almost 15N with respect to the forces recorded during squatting, from 6.8(0.6)N for squatting to 21.6(0.6)N. Higher torques are also recorded in stooping movements, with an increase of 1.1 Nm, from a minimum value of −1.1(0.02) to −2.2(0.17) Nm. Indeed, the subject’s pose between 40 and 60% of the cycle is the most challenging for the SAM, with the exoskeleton’s and the lower leg’s coordinate origin frames recording their highest drifts, reported in the 3rd row of Figure 11 and Table 7. For the squatting motion, the offsets reported in 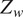 and 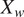 have a higher absolute difference (9.4 and 10.3, respectively) with respect to stooping movements.

The MIL simulation results are also divided for *squat* and *stoop* movements. In Figure 12 we present joint solutions from the IK simulation. The plots show the simulated values with standard deviation from 0 to 100% of the performed movement. In addition, for the joints that reach saturation, a thick horizontal orange bar is added. Between 10 and 20% the two joints 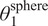 and 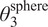 completely saturate at −10° for both squatting and stooping. During the rest of the cycle, 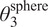 jumps to its opposite saturation angle 10° while 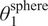 values have a burst of movement but remain at −10°. Recalling the kinematic arrangement in Figure 4, the saturation of two revolute ( 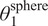 and 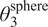 ) influences the parasitic torques on the leg straps.

In Table 10 we report all the time percentiles where joints saturate and 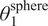 prove to be in saturation for 63 and 57% of the cycle for squatting and stooping for S1. Reachability maps, in Figure 13a,b, shown in a solid color scale only the voxels that contain EE and HT workspace points. Transparent red voxels contain only HT workspace points, the color map shows the corresponding Reachability values. Capability maps show only the voxels containing HT trajectory and EE workspace points. The color map shows the maximum and minimum Capability values for every run and repetition. Figure 13c,d show in Cartesian space, the trajectory points, and Capability values. The voxels with lower Capability (in the range 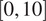 for stooping and for squatting movements) are those that contain trajectory points from the central part of the movement cycles. In fact, as Figure 11c,d, higher forces and torques appears in that part. Indeed, in Figure 13a,b, the same voxels have low Reachability value (in the range between 0.2 and 0.4). Figures 14 and 15 show the Capability and Reachability values versus the percentage of the total voxels.

## 5 Discussion

The software tool presented in this work is designed to analyze, through kinematics simulations, a rigid bodies’ chain composed by human lower limbs H and an occupational exoskeleton E. Specifically, this tool is to identify the source of parasitic forces and torques that are recorded at the attachment points, those sources are searched in critical kinematic configurations. The simulation tool is, then, qualitatively assessed on XoTrunk and its user kinematics.

*Methodological Contributions* This work presents a human MIL approach to describe the capabilities of an exoskeleton’s kinematic chain and its robustness to different fits (*inter-subject variability*) and braces movements over the body (*intra-subject variability*). State of the art simulation tools similar to ours, like Cempini et al. (2013) require a specific mathematical description of the device, and it can synthesize a kinematic chain configuration. Our approach is intended to investigate the performance of any rigid wearable device and it uses standard tools from robotics to assess existing kinematic chains while a design solution is not provided to improve design and reduce the parasitic forces. Indeed, our preliminary qualitative results suggest that parasitic force and torques at the leg anchor point, Figure 11c,d are mostly associated to two specific joints ( 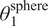 and 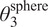 in Figure 12). Additionally, in this work, Reachability and Capability index are introduced for exoskeletons for the first time. Reachability is calculated from the intersection of LW and EW. Capability is calculated from the pose following error of EE with respect to HT trajectory. The use of spatial maps with the representation of Reachability and Capability can help designers to identify critical links and joints that are contributing to misalignments. In fact, a voxel that results in low Reachability index or low Capability is likely to contain exoskeleton’s configurations that creates undesired forces. If a point of 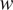 is in a voxel with a high Reachability, a large error might be the result of a singularity in the EE. As an example the reader may refer to Figure 15. in that case, the simulation shows that all subjects and movements have the most voxels within the range of 60–80% of their maximum Reachability and low Capability values for the central part of the movement cycle (yellow voxels in the right end of maps in Figure 13c,d). Indeed, the joints 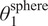 and 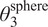 are saturated up to 90% and 23% of the movement cycle for S3, as shown in Table 10. As a result of this analysis, a designer might consider to address this issue by replacing in the simulation the spherical joint with a new with a broader RoM. A new simulation will evaluate the effects of this suggested solution without the need to create a new prototype. The new simulation could also suggest to re-arrange the kinematics of the misalignment compensation mechanism with a different joints placement, because of the minor saturation events occurring for joints 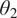 and 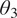 .

*Experimental Contribution* To validate the proposed method, we used it to perform an analysis of the exoskeleton XoTrunk. The qualitative results show the influence of different anthropometrics and different movements on exoskeleton performance. In fact, subject S3 has greater hip and thigh width but a shorter body rise with respect to the other subjects (shown in Table 6). Those differences result in more severe fit problems, bigger 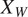 drift, and, consequently, higher parasitic reaction forces and torques (32.3 N and 4.6 Nm for stooping and 27.5 N and 3.2 Nm for squatting). In fact, bigger 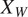 drift implies a larger distance of the exoskeleton’s actuator ICR with respect to hip joint along 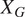 direction. The results suggest that even for smaller bending angles, drifts and anthropometrics creates undesirable parasitic forces. In fact, S2 performs the stooping movement with the highest bending angle ( 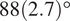 ) but this results in the lowest parasitic forces and torques for that movement with respect to the other subjects (19.2 N and 2.8 Nm). This result suggests that the implemented R–R–S mechanism is not sufficient to compensate all the misalignments introduced by the anthropometric variability. In addition, the method show also the effects of different movements on the same subject. In fact, there are notable differences in parasitic reactions in every stoop with respect to squat. Table 8 and Figure 11 show that for every subject the stoop movements create higher values of reaction forces and torques, an average of 15 N and 2.7 Nm more in stoops. To conclude, during the experiment the XSens system was used to record the relative motion of the belt with respect to the desired attachment point on the waist. To the author's knowledge, this is the first time that this use was proposed for wearable robots. The system proved to be effective in recording the relative motion induced by the parasitic forces that loosen the braces, reconstructing the drifts in *X* and *Z* planes. The data collected is of paramount importance for the simulation, as it is part of the inputs of the model, along with the motor angular position and hip joint angles.

### 5.1 Limitations and Future Works

The work presented in this manuscript is intended as a first step toward a more complete simulation. In fact, different points to be addressed are listed.

*Quantitative assessment* In “Experimental design and protocol” section, we describe the protocol to validate the MIL simulation, however, no quantitative validation of DK results is provided even if the IK output has preliminarily proved to be in accordance with the recorded output effects of kinematic errors. To address this issue, the model will be expanded to provide computed forces and torques at the fixation points. Thus allowing to compute a formal error with respect to the experimental data.

*Test protocol update* Few subjects with little anthropometric variability were used, which limits the generalizability. To avoid adding dynamic effects the execution speed of the squatting and stooping movements should be imposed to be the same for all the subjects and all the repetitions. The braces should be checked in every repetition. In a future experimental campaign, we will assess the model with more subjects. Inclusion criteria will be defined to create a relevant subject group. This group should effectively represent the exoskeleton’s targeted users population, workers with an occupation dealing with un-ergonomic manual material handling.

*Sensors* The *F*/*T* sensor was not accounted for affecting force and torque readings; it alters the original kinematics and add inertia. We will search for alternative *F*/*T* sensors reduced in dimension and weight. This will be possible thanks to the recorded data of this work, allowing a better sizing of the new sensors. Lastly, it was not possible to record the relative motion of the belt in all directions due to drifts in the IMU’s readings. In fact, Xsens Fusion software engine filters and calibrate dynamic offsets, however, it was not possible to have the same for the Prop. Two different solutions can be investigated: implementation of an offline calibration algorithm or changing to any optical markers measurement systems if it is possible to avoid occlusion.

*Model update* The exoskeleton’s kinematic model will be updated to include the shoulder braces. In addition, we will extend the Xsens sensing ability to record shoulder and leg braces fit and drifts. Currently, Capability is computed from the norm of pose error, however, the map may be more informative if angular and position errors could be retrieved separately (instead of having just a single value of pose error). In addition, we will evaluate to add recorded data compliance checks, to avoid introducing possible errors (e.g., continuity constraints, exceeding RoMs).

## 6 Conclusions

The kinematics and the proper alignment with the corresponding biological joints are a central aspect of exoskeleton’s design. Poor alignment, along with the resulting undesirable forces, negatively influence physical comfort and might compromise users’ acceptance. Sources of misalignments include (i) exoskeleton fitting problems due to user anthropometry (*inter-subject* variations) and device migration during use (*intra-subject* variations), (ii) the presence of compliance in braces, and (iii) several passive DoFs. These pose the need for improvements and a great challenge for designers to identify issues in the exoskeleton’s kinematic (e.g., passive joints with an insufficient ROM). This paper describes a MIL approach that aims at supporting the design and assessment of the kinematic structures of exoskeletons, such as the R–R–S misalignment compensation mechanism, considering the aforementioned issues. The first methodological contribution is a tool that creates a simulation environment using XoTrunk occupational exoskeleton’s kinematics and several variables recorded from real users. These variables are: kinematic and motion data, exoskeleton’s fit data, the relative motion of the belt attachment, parasitic forces and torques recorded from subjects during squatting and stooping motion. The output of the simulation is data, that is interpreted to discover saturations (i.e., Boundaries Singularity) in joints configurations that could give rise to parasitic forces and torques. The second methodological contribution is the result visualization. Results are reported in both time and Cartesian space (i.e., Reachability and Capability maps) plots, that show the kinematic joints configurations that saturate for every subject during their movements. Reachability map can show if the exoskeleton’s and user’s Primary workspaces overlap. Capability map can show in a compact way the local dexterity of the exoskeleton over a preferred direction (i.e., thigh attachments trajectories). In addition, the experimental contribution qualitatively assess the methodology proposed with a preliminary testing campaign. Therefore, we obtain a valuable insight to update the design to remove the sources of misalignments. This tool can be helpful in different scenarios of the design process of an exoskeleton. It can be used in the involvement of the final users as recommended in the UCD approach. The tools can output visual 3D reconstruction of the movements performed by the exoskeleton combined with the body and, therefore, help the users understand how the exoskeleton works to exploit its possibilities at their best. The tool can save time to designers as new kinematics arrangements can be tested or to assess new and innovative exoskeleton designs without building a prototype. The assessment could lead to test the robustness of the exoskeleton to perturbation in state-space variables, exploiting the power of Reachability and Capability to show the effects. To achieve that, the model needs to be expanded to be able to calculate internal and reaction forces resulting from the kinematic arrangement. Afterward we will quantitatively assess the new model with a larger group of test subjects with and updated testing protocol, to overcome the issues encountered in this work.

## Data Availability

The data that support the findings of this study are available from the corresponding author, M. Sposito, upon reasonable request.

## Appendix A. Direct and IKs

A kinematic chain is defined as a series of rigid body  , linked together by a prismatic joint (pure translation), revolute joint (pure rotation), or rigidly coupled. The pose in space of a rigid body  is completely defined by the rotation and distance of its reference frame  referred to a global reference frame  . A convenient notation to describe a kinematic chain is the use of Denavit–Hartenberg (DH) parameters, that allows to define any point of the chain as the composition of reference frames transformations through the chain’s several rigid bodies to a global refence. Any point of a kinematic chain *K* can be described in DH by a series of coordinate body frames transformations  in robot joints variables  and link dimensions in a vector *p*. A homogeneous transformation matrix *T* contains both  and *p*.

(4)

A matrix *T* is composed by all the joint variables and dimensions of the links that describes. A linear composition of homogeneous matrix represent a transformation composed by subsequent transformations. To compute Direct Kinematic of the EE of a robot, given the joints configuration  , it is enough to know the matrix T for every link from the global frame  to the EE frame

(5)

With DK it is possible to build the workspace in Cartesian space starting from the robot’s joint configuration space. Finding a joint configuration given a point in space is possible only when the point in space is in the dextereous workspace of the robot, usually there are multiple solution depending on robot redundancy. For non-standard robot configurations IK solution are computed via numerical methods. The method used in this work implements the IK algorithm *BFGS Gradient projection* available in *Matlab Robotic Toolbox*, for further information see (Goldfarb, 1969).

## Appendix B. Movement Reconstruction from Quaternions

Xsens Fusion Engine is a software built-in in the Xsens Analyze suite, the Fusion engine bases its calculation to reconstruct body movements on inertial sensors reading. In this way, any body segment has an orientation, acceleration, and velocity. To represent a three-dimensional rotation we need to define a rotation axis and a rotation angle. The unit quaternion can represent these information in a compact and convenient way and is an alternative to rotation matrix notation. A rotation around *r* of  can be representend by *Q* or its vector notation, Equation (7)

(6)

(7)

A composition of multiple rotations can be obtained composing the relative quaternions through the * operator defined as

(8)

As for rotation matrices, a vector *x* in a reference frame *B* can be expressed in the frame *G* through composition of quaternion

(9)

where the inverse of a quaternion  is  .

To reconstruct the movement of a point in any reference frame *B* referred to a world frame *G*, it is sufficient to know the quaternion  . For further informations, see (Kuipers, 1939).
